# Modulating the gut microbiome to enhance cancer immunotherapy: a systematic review and Meta-Analysis of probiotics and FMT as adjuncts

**DOI:** 10.1186/s12885-026-15655-6

**Published:** 2026-01-28

**Authors:** Saidi Hu, Chenchen Luo, Siran Wan, Shunhong Zhang, Nian Li, Guangyue Liu, Lin-Yong Zhao

**Affiliations:** 1 Department of Stomatology, Ya’an people’s Hospital, Ya’an, China; 2 Department of outpatient chengbei, the Affiliated Stomatological Hospital, Southwest Medical University, Luzhou, China; 3 Department of Gynaecology and Obstetrics, Ya’an people’s Hospital, Ya’an, China; 4 Department of Cardiology, Pangang Group General Hospital, Panzhihua, China; 5 Department of Traditional Chinese Medicine, Panzhihua Central Hospital, Panzhihua, China; 6 Department of Anesthesiology, West China Hospital, Sichuan University, Chengdu, China; 7 Department of General Surgery & Laboratory of Gastric Cancer, State Key Laboratory of Biotherapy / Collaborative Innovation Center of Biotherapy and Cancer Center, West China Hospital, Sichuan University, Chengdu, China; 8 Gastric Cancer Center, West China Hospital, Sichuan University, Chengdu, China

**Keywords:** Cancer, Immune checkpoint inhibitors (ICIs), Gut microbiota, Probiotics, Fecal microbiota transplantation (FMT), Meta-analysis

## Abstract

**Background:**

Although the gut microbiota modulates cancer immunotherapy efficacy and interventions such as probiotics and fecal microbiota transplantation (FMT) may enhance antitumor response, clinical evidence remains controversial, prompting this meta-analysis to evaluate their impact on immune checkpoint inhibitors (ICIs) outcomes.

**Methods:**

A systematic search was conducted in PubMed/Medline, Embase, and trial registries (ClinicalTrials.gov, chictr.org) for relevant records up to August 2025. ORR and DCR defined as primary composite endpoints, and PFS/OS as secondary endpoints. Data were synthesized using random-effects models to calculate pooled estimates for ORR, DCR, and hazard ratios (HRs) for PFS and OS.

**Results:**

A total of 22 studies involving 3,274 patients were included. The pooled analysis demonstrated that probiotic intervention was associated with a reduced risk of progression or death, as evidenced by improved PFS (pooled HR = 0.63, *P* < 0.0001) and OS (pooled HR = 0.53, *P* < 0.00001) in cancer patients receiving ICIs. Similarly, interventions using either probiotics or FMT were associated with an increased ORR (pooled OR = 1.62, *P* = 0.006) and showed a trend toward improved DCR (pooled OR = 1.74, *P* = 0.12).

**Conclusion:**

This meta-analysis supports that both probiotics and FMT, as adjunctive therapies, are associated with enhanced efficacy of cancer immunotherapy. Probiotics, in particular, are supported by more robust evidence and demonstrate more consistent effects. Future large-scale, rigorous clinical trials are warranted to advance the development of personalized and precise microbiota-based interventions.

**Supplementary Information:**

The online version contains supplementary material available at 10.1186/s12885-026-15655-6.

## Introduction

Immune checkpoint inhibitors (ICIs), including programmed cell death protein 1 (PD-1)/programmed death-ligand 1 (PD-L1) and cytotoxic T-lymphocyte-associated protein 4 (CTLA-4) inhibitors, have significantly improved outcomes in advanced cancers [1]. However, their efficacy remains limited—for example, in melanoma, the 3-year progression-free survival (PFS) rate is only 34.1–36.6% [2]. This limitation is largely attributable to innate or acquired resistance mechanisms, which preclude clinical benefits in most patients [3]. Mechanistic insights into the tumor microenvironment (TME) [4, 5] and the gut microbiota have highlighted the importance of gut microbes in modulating host immunity and cancer treatment outcomes [6–8]. Clinical evidence indicates that the gut microbiota critically modulates response to immunotherapy, as demonstrated by Bacteroides species enhancing CTLA-4 inhibitor efficacy through an interleukin-12 (IL-12)-dependent T helper 1 (TH1) immune response [6]. Moreover, multiple studies have consistently demonstrated significant structural differences in gut microbiota between responders and non-responders to immunotherapy [9, 10], suggesting that microbiome-targeted interventions may help overcome treatment resistance.

Notably, the immunomodulatory influence of the microbiota extends beyond the gut. For instance, some beneficial bacteria enriched in ICI responders originate from the oral cavity [11], and the salivary microbiome may predict outcomes in oral cancers [12]. These findings underscore the systemic impact of the microbiota and position the targeted modulation of the gut ecosystem as a promising strategy to enhance immunotherapy. Among various approaches (e.g., diet, prebiotics, engineered microbes), probiotics and fecal microbiota transplantation (FMT) have emerged as the most directly translatable and clinically investigated interventions.

Probiotics primarily function by introducing specific beneficial strains that gently modulate both local intestinal and systemic immune environments through the bacteria themselves or their metabolites. Taking the probiotic formulation Clostridium butyricum MIYAIRI 588 (CBM588) as an example, it produces butyrate, which enhances antitumor immunity by epigenetically upregulating T cell activity and facilitating cluster of differentiation 8 positive T Cells (CD8⁺ T cells) activation, thereby improving the TME and the efficacy of PD-1 inhibitors [13]. Clinical researches [14–18] on CBM588 are accumulating, and studies [10, 19–25] on other probiotics are also advancing. While traditional probiotic preparations typically contain only single or limited bacterial strains—making it difficult to fully replicate the functional diversity of the gut microbiota—FMT aims to achieve a “complete reset” of the intestinal ecosystem. Relevant studies have shown that by transplanting the complete microbiota from healthy donors [26–28] or immunotherapy responders [29–33], FMT can restore the compositional and functional diversity of the patient’s gut microbiota, thereby more comprehensively reshaping immune homeostasis. Nevertheless, this approach faces a series of ethical challenges and issues of inconsistent efficacy.

To systematically compare the efficacy differences between probiotics and FMT in cancer immunotherapy, we performed a meta-analysis to evaluate the impact of these two interventions, when combined with ICIs, on key efficacy outcomes in patients with advanced cancer. These outcomes include objective response rate (ORR), disease control rate (DCR), overall survival (OS) and PFS. By synthesizing existing clinical evidence, we aim to provide clarity for clinical decision-making and guide the future development of precise, personalized microbiome therapies.

## Methods

The conduct and reporting of this study adhered to the Preferred Reporting Items for Systematic Reviews and Meta-Analyses (PRISMA 2020) guidelines. The protocol for this meta-analysis has been registered in PROSPERO (Registration number: CRD420251165733). As the research utilized exclusively de-identified patient data, it was exempt from ethical review board approval and the need for individual patient consent, in accordance with pertinent regulations.

### Literature search strategy

We conducted a systematic search in PubMed/MEDLINE, Embase, and Web of Science for studies published from database inception up to August 2025. A comprehensive search strategy was developed, integrating controlled vocabulary (e.g., MeSH, Emtree) and free-text keywords. The strategy was built around three core concepts: (1) neoplasms (2), specific gut-microbiota-modulating interventions (probiotics and FMT), and (3) cancer immunotherapy. The detailed search strings for each database are provided in Supplementary Material (Supplementary Table S1). The literature search was restricted to formally published works in the English language. To ensure comprehensiveness, the search was supplemented by screening clinical trial registries (ClinicalTrials.gov, WHO chictr.org) and manually screened the reference lists of all included studies and relevant systematic reviews.

### Eligibility criteria

Two investigators (S.H. and C.L.) independently performed the study selection in a two-step process. First, titles and abstracts were screened for relevance. Subsequently, the full texts of the remaining articles were thoroughly assessed for eligibility based on the pre-defined criteria. All disagreements encountered during this process were first discussed between the two primary reviewers. If a consensus could not be reached, the final decision was made by a third investigator (S.Z.).

#### Inclusion criteria

Studies were included for the following criteria:

1. Study Population: Adult patients (≥ 18 years) diagnosed with advanced/metastatic cancer who were receiving treatment with ICIs, either as monotherapy or in combination regimens.
2. Study Design: Clinical studies of any design that investigated the efficacy of microbiome-modulating interventions (probiotics or FMT) in the above patient population. Eligible designs included randomized controlled trials (RCTs), prospective or retrospective cohort studies, single-arm interventional trials, and non-randomized comparative studies.
3. Interventions: Studies must have involved a defined microbiome-modulating intervention, specified as the use of probiotics (any species, formulation, or dosage) or FMT.
4. Outcomes of Interest: Studies must have reported at least one of the following efficacy endpoints:
  Primary composite endpoints: ORR, defined as the proportion of patients achieving a complete response (CR) plus partial response (PR); and DCR, defined as the proportion achieving CR + PR + stable disease (SD). CR, PR, and SD were considered only as components of these composite endpoints.
  Secondary endpoints: PFS, defined as the time from treatment initiation to disease progression or death from any cause. OS, defined as the time from treatment initiation to death from any cause.
5. Data Availability: Studies must have provided complete and reliable data, reporting 95% confidence intervals (CIs) or providing statistics (e.g., event counts, hazard ratios, P-values) that allowed for calculation.

#### Exclusion criteria

Studies were excluded for the following criteria:

1. Irrelevant Population: Studies not involving adult patients (≥ 18 years) with advanced/metastatic cancer or not evaluating treatment with ICIs.
2. Ineligible Study Design or Publication Format: Studies were excluded if they met any of the following criteria: being non-clinical studies (e.g., animal or in vitro research), non-original research (e.g., reviews, meta-analyses), conference abstracts without full-text, non-peer-reviewed reports, employing an intervention that was not a clearly defined probiotic or FMT regimen, or involving combined interventions where the independent effect of the probiotic/FMT component could not be isolated.
3. Inadequate data: Studies were excluded if they did not report at least one pre-specified efficacy endpoint (ORR, DCR, PFS, or OS), reported outcomes only graphically without numerical data, reported only component outcomes (e.g., CR, PR, SD) without the composite endpoints ORR or DCR (unless calculable), or had missing or insufficient statistical data (e.g., lacking event counts, HRs, CIs, or P-values) to calculate effect estimates.
4. Methodological limitations or Inadequate Reporting: Studies were also excluded if they were single-arm or cohort studies with fewer than 10 patients in the intervention group; if they were retrospective studies with over 50% missing data for key variables needed for adjusted analysis; or if they had overlapping patient cohorts (in which case only the most comprehensive or recent publication was retained).

### Data extraction and quality assessment

Two investigators (S.H. and S.W.) independently extracted data, including the first author, publication year, study design, sample size, intervention type, tumor type, and outcome measures. The methodological quality was assessed independently by two other investigators (N.L. and G.L.). For RCTs, the Cochrane Risk of Bias 2.0 tool (ROB 2.0) was used. For the included single-arm, non-randomized interventional studies evaluating FMT, no dedicated tool exists. Given their interventional nature, the Risk Of Bias In Non-randomized Studies - of Interventions (ROBINS-I) tool was applied to assess bias arising from the lack of a randomized control group. Additionally, the Newcastle-Ottawa Scale (NOS) was used to score the quality of cohort studies, with a score of ≥ 7 indicating high quality, 5–6 indicating moderate quality, and < 5 indicating low quality.

### Statistical analysis

All statistical analyses were performed using R software (version 4.5.1) and Review Manager (version 5.4). Dichotomous variables (e.g., DCR, ORR) were expressed as pooled proportions with 95% CIs to describe absolute event rates. It should be noted that this descriptive synthesis incorporated data from both single-arm and two-arm studies; therefore, these pooled proportions do not represent comparative effectiveness. For the analysis of comparative effectiveness, time-to-event variables (e.g., PFS) were expressed as pooled HRs, and dichotomous outcomes for comparative analysis as pooled ORs, each with 95% CIs. The pooled HRs and ORs in this meta-analysis were derived exclusively from two-arm study designs (e.g., cohort studies and RCTs) to ensure that the effect estimates were based on direct within-study group comparisons. A random-effects model was applied to accommodate significant heterogeneity, as assessed by *τ*² and *I*² statistics, based on our pre-defined criteria (Cochran’s Q-test *P*-value < 0.10 and/or an *I*² statistic ≥ 50%). The degree of statistical heterogeneity was assessed using the I² statistic, classified as follows: low (*I*² < 25%), moderate (25% ≤ *I*² < 50%), high (50% ≤ *I*² < 75%), or very high (*I*² ≥ 75%). To explore potential sources of heterogeneity, pre-specified subgroup analyses were conducted. Publication bias was evaluated by visual inspection of funnel plot symmetry, supplemented by Begg’s rank correlation test and Egger’s linear regression test; a *P*-value < 0.05 in Egger’s test was considered suggestive of potential publication bias. A two-sided *P*-value < 0.05 was considered statistically significant for all analyses.

## Results

### Search results

**Fig. 1:**
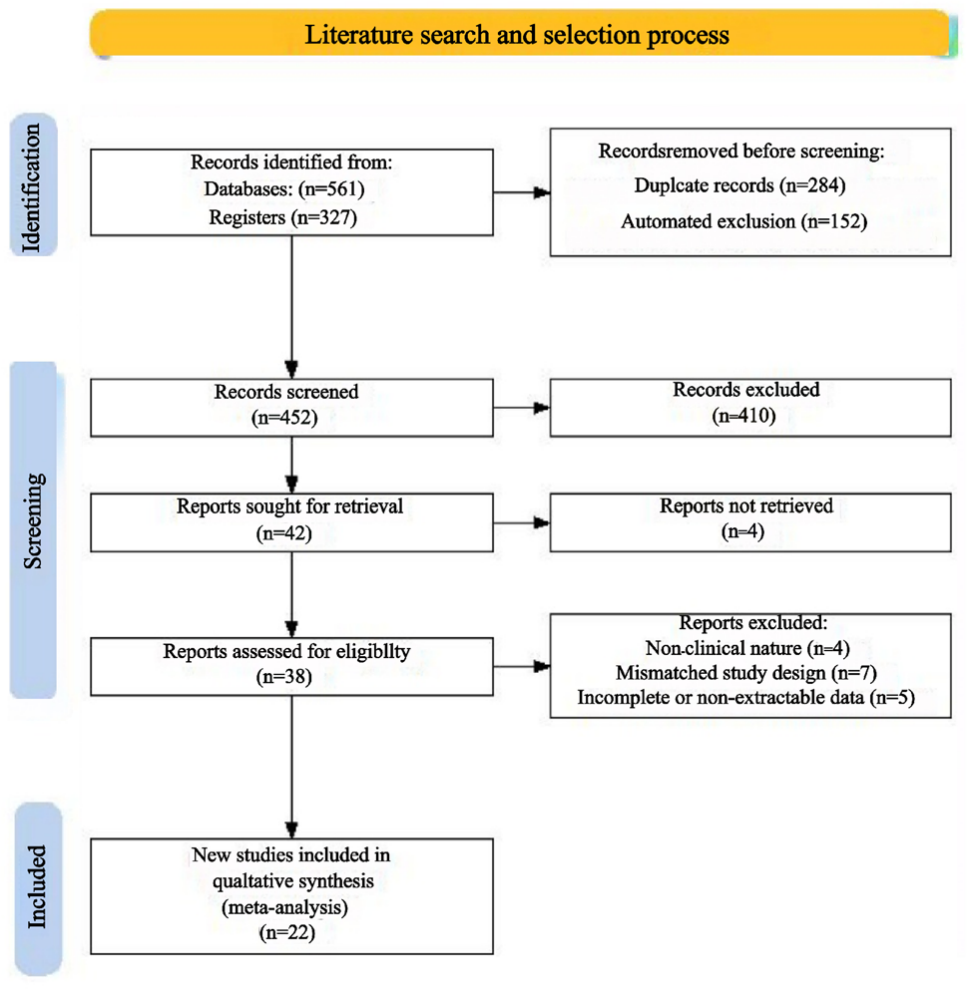
Literature search and selection process. Identification: Databases searched included PubMed/MEDLINE, Embase, and Web of Science. Trial registers searched included ClinicalTrials.gov and the WHO ICTRP. Automated Exclusion: This step involved filtering records based on predefined irrelevant keywords (e.g., non-cancer topics) in titles/abstracts. Screening (Records Excluded). Records were excluded if published in a non-English language and their relevance could not be assessed

A total of 888 records were identified through databases and registers (561 from databases and 327 from registers). After the removal of 284 duplicates, an additional 249 records were excluded through an automated screening process: 249 were automatically filtered out as clearly irrelevant based on title/abstract keywords (e.g., non-cancer topics). The remaining 452 records underwent title and abstract screening. Following the exclusion of 410 records at this stage, 42 full-text articles were sought for retrieval. 4 articles could not be retrieved, leaving 38 articles for full-text eligibility assessment. After detailed evaluation, 16 articles were excluded for not meeting the predefined criteria, primarily for the following reasons: (1) non-clinical nature; (2) mismatched study design; (3) incomplete or non-extractable data. Ultimately, 22 studies were included in the qualitative systematic review and quantitative meta-analysis. (Fig. 1)

### Quality assessment

A total of 22 studies were included in this analysis. Their methodological quality was assessed using the ROB 2.0, ROBINS-I, and NOS tools, as detailed in the Methods. The results indicated that the overall evidence base was of moderate overall credibility, limited by the methodological constraints of the included observational and single-arm studies. Specifically: 3 randomized controlled trials (RCTs, 2 on probiotics and 1 on FMT) were rated as having “some concerns”, with limitations mainly related to the implementation of blinding and the objectivity of outcome measurement; 7 non-randomized single-arm interventional studies (all on FMT) were rated as having “moderate risk of bias”, primarily due to confounding bias resulting from the lack of control groups; and 12 cohort studies (all on probiotics) were of moderate to high quality, with two being rated as high quality.

To integrate these findings into an overall judgment, we adopted a conservative framework that prioritized the limitations identified by the most stringent applicable tool. The “some concerns” in RCTs and the inherent “moderate risk of bias” in single-arm studies (due to design limitations) preclude a judgment of high overall certainty. However, the generally moderate-to-high quality of the observational cohort studies provides a consistent signal. Consequently, we conclude that the current evidence is of moderate credibility, sufficient to suggest a potential association or effect but underscoring the need for more rigorously designed RCTs to establish causal inference (Supplementary Table S2A-C).

### Characteristics of included studies

This meta-analysis included a total of 22 studies, comprising 12 cohort studies, 3 RCTs, 7 non-randomized single-arm interventional studies. These studies were primarily conducted in Japan (7 studies), China (4 studies), the United States (4 studies), and Canada (4 studies), among other countries, and encompassed various cancer types including NSCLC (9 studies), melanoma (4 studies), renal cell carcinoma (RCC) (3 studies), and solid tumors (3 studies). The median patient age was approximately 65 years, with males accounting for 72.1% of the study population. Interventions included probiotic preparations (14 studies) [10, 14–25] and FMT (8 studies) [26, 28–34]. All included studies reported data on at least one of the four efficacy endpoints of interest, which are presented in order of priority: ORR and DCR served as the primary composite endpoints, followed by PFS and OS as secondary endpoints. The sample sizes ranged from 10 to 482 patients, and the overall characteristics were reasonably distributed, demonstrating good representativeness. (Table 1)

**Table 1:** The characteristics of included studies. Studies are grouped by intervention type and sorted alphabetically by first author’s surname and then by publication year within each group

Author and Year	Country/Region	Study Design	Cancer Type	Study Period	Trial Registration NO.	ICIs treatment Regimen	Participants (EG/CG)			Intervention Type/ (Donors)	Outcome Measures
							Number	Median Age	Male		
Dizman et al.2022 [15]	USA	RCT	mRCC	04/2019-12/2020	NCT03829111	Nivo + Ipi	19/10	66/64	13/8	CBM588	ORR; DCR; PFS
Ebrahimi et al.2024 [17]	USA	RCT	mRCC	11/2021-03/2023	NCT05122546	Nivo + Cabozantinib	20/10	68/60	15/5	CBM588	ORR; DCR; PFS
Luo et al.2024 [10]	China	Prospective cohort study	NSCLC	03/2019-09/2022	NA	Anti-PD−1/L1 mono or Anti-PD−1/L1 + CT/Ais or Anti-PD−1/L1 + CT + Ais	11/63	59/56	4/24	Bio-Three Tablets	ORR; PFS; OS
Miura et al.2021 [20]	Japan	Retrospective cohort study	NSCLC	01/2016-07/2018	NA	Nivo/Pembro mono	14/286	65	226	Probiotics (unspecified)	ORR; OS
Morita-ID et al.2024 [18]	Japan	Retrospective cohort study	NSCLC	12/2015-05/2018	NA	Anti-PD−1/L1 mono	93/389	68/69	68/312	CBM588; non-spore-forming bacteria	ORR; DCR; PFS; OS
Morita-ICD et al.2024 [18]	Japan	Retrospective cohort study	NSCLC	12/2018-12/2020	NA	Anti-PD−1/L1 + CT	77/368	68/69	19/61	CBM588; non-spore-forming bacteria	ORR; DCR; PFS; OS
Spencer et al.2021 [21]	USA	Retrospective cohort study	Melanoma	04/2015-01/2019	NA	Anti-PD−1/Anti-CTLA4 mono or combination therapy	49/109	64	27/67	Probiotics (unspecified)	DCR; PFS
Svaton et al.2020 [19]	The Czech Republic	Retrospective cohort study	NSCLC	2015–2019	NA	Nivo	6/218	67	133	Lactobacillus	PFS; OS
Takada et al.2021 [22]	Japan	Retrospective cohort study	NSCLC	01/2016-09/2018	NA	Nivo/Pembro mono	32/262	67	25/208	BIOFERMIN; LAC-B; CBM588; ARLAB	PFS; OS
Takada et al.2022 [23]	Japan	Retrospective cohort study	NSCLC	01/2016-09/2018	NA	Nivo/Pembro mono	32/261	NA	233	BIOFERMIN; LAC-B; CBM588; ARLAB	PFS; OS
Tomita et al.2020 [14]	Japan	Retrospective cohort study	NSCLC	01/2016-05/2019	NA	Nivo/Pembro/Atezo mono or combination therapy	37/69	68/67	33/66	CBM588	ORR; DCR; PFS; OS
Tomita et al.2023 [16]	Japan	Retrospective cohort study	NSCLC	01/2019-8/2022	NA	Pembro/Atezo + CT or Atezo + Ais+CT or Nivo + Ipi+CT	45/55	67/66	33/39	CBM588	PFS; OS
Tong et al.2024 [24]	China	Prospective cohort study	Lung cancer	06/2021-12/2022	NA	Anti-PD−1/L1 + CT/CRT/Ais or Anti-PD−1/L1 + CT + Ais or Anti-PD−1/L1 + cell therapy	71/182	60/63	59/153	Probiotics (unspecified)	ORR; DCR; PFS
Wang et al.2024 [12]	China	Retrospective cohort study	HCC; CRC; GC	03/2019-07/2022	NA	Anti-PD-1 + Ais	79/263	60	275	Probiotics (unspecified)	ORR; PFS; OS
Baruch et al. 2017 [31]	Israel	Phase I Single-arm study	Melanoma	06/2018-03/2019	NCT03353402	Nivo	10	66	7	FMT (ICIs responders)	ORR
Davar et al. 2017 [30]	USA	Phase II Single-arm study	Melanoma	06/2018-01/2020	NCT03341143	Pembrob	15	61	14	FMT (ICIs responders)	ORR
Duttagupta et al. 2024 [33]	Canada	Phase II Single-arm study	Solid tumors	11/2021-04/2024	NCT04951583	Pembro/Nivo + Ipi	20	56	13	FMT (ICIs responders)	ORR
Fernandes et al. 2020	Canada	Phase I Single-arm study	mRCC	11/2019-09/2020	NCT04163289	Ipi+ Nivolumab	10	59.5	8	FMT (Healthy people)	ORR
Hadi et al. 2025 [34]	Canada	Phase I Single-arm study	Melanoma	06/2019-09/2021	NCT03772899	Pembro/Nivo	20	75.5	12	FMT (Healthy people)	ORR; DCR; PFS; OS
Kim et al. 2024 [32]	Korea	Single-arm study	Solid tumors	02/2019-08/2020	NCT04264975	Pembro/Nivo	13	60	10	FMT (ICIs responders)	ORR
Spreafico et al. 2023 [26]	Canada	Phase II/III RCT	Solid tumors	12/2018-12/2020	NCT03686202	Nivo/Pembro mono or Nivo + Ipi	26/10	70/68	19/7	MET4 (Healthy people)	ORR; DCR
Zhao et al.2023 [29]	China	Phase II Single-arm study	MSS-mCRC	05/2021-01/2022	ChiCTR2100046768	Tisle+ Fruq	20	62	18	FMT (ICIs responders)	ORR; DCR; PFS

*Abbreviations*: *AIs* Anti-angiogenesis Inhibitors, *Anti-CTLA-4* Anti-cytotoxic T-Lymphocyte-Associated Protein 4, *Anti-PD-1* Anti-programmed Cell Death Protein 1, *Anti-PD-L1* Anti-programmed Death Ligand 1,*ARLAB* Antibiotic-resistant Lactic Acid Bacteria, *Atezo* Atezolizumab, *CBM588* Clostridium butyricum MIYAIRI 588, *CRC* Colorectal Cancer, *CRT* Chemoradiotherapy, *CT* Chemotherapy, *DCR* Disease Control Rate, *FMT* Fecal Microbiota Transplantation, *Fruq* Fruquintinib, *GC* Gastric Cancer, *HCC* Hepatocellular Carcinoma, *ICIs* Immune Checkpoint Inhibitors, *Ipi* Ipilimumab, *mRCC* Metastatic Renal Cell Carcinoma, *Mono* Monotherapy, *MSS-mCRC* Microsatellite Stable Metastatic Colorectal Cancer, *NA* Not Available, *Nivo* Nivolumab, *NSCLC* Non-Small Cell Lung Cancer, *ORR* Objective Response Rate, *OS* Overall Survival, *Pembro* Pembrolizumab, *PFS* Progression-Free Survival, Tisle, Tislelizumab

### Objective response rate (ORR)

**Fig. 2:**
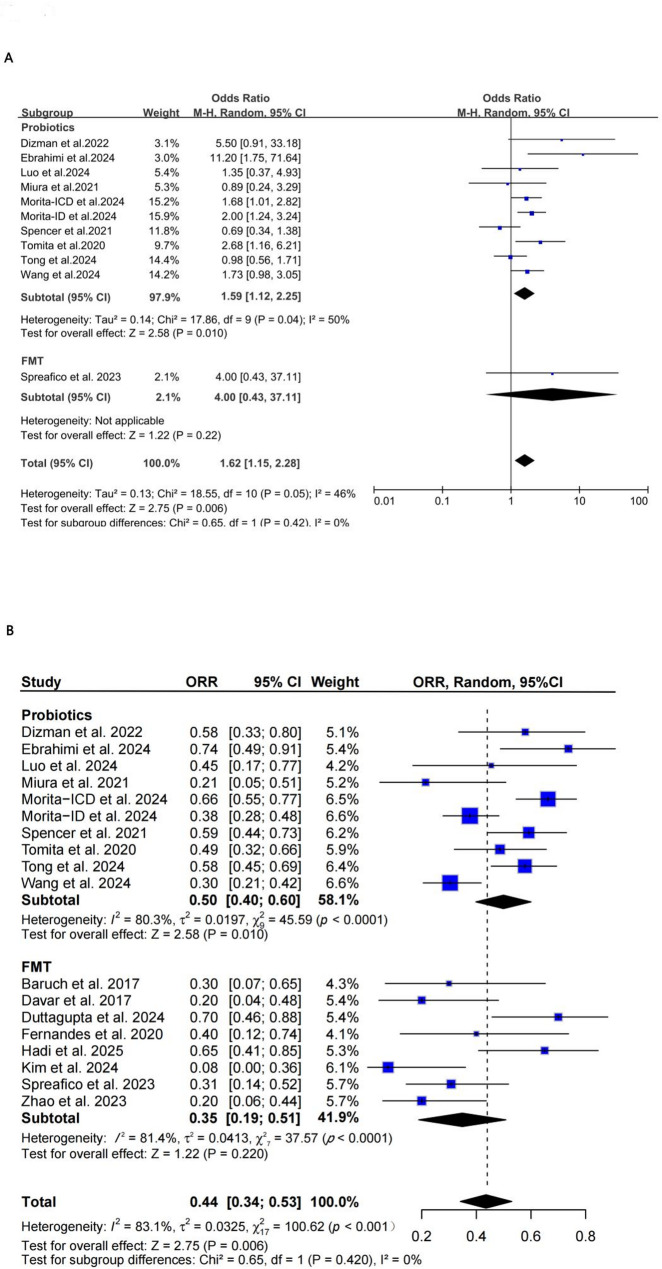
**A**: Forest plot of comparative analysis: Pooled OR for ORR in patients receiving ICIs with probiotics or FMT versus control (based on two-arm studies only). **B**: Forest plot of descriptive analysis: Pooled event rates (proportions) of ORR in patients receiving ICIs with probiotics or FMT (including both single-arm and two-arm studies), Note: These pooled proportions are derived from both single-arm and two-arm studies and should not be interpreted as measures of comparative effectiveness between interventions. Studies are grouped by intervention type and sorted alphabetically by first author’s surname and then by publication year within each group. Abbreviations: FMT, Fecal Microbiota Transplantation; ICIs, Immune Checkpoint Inhibitors; OR, Odds Ratio; ORR, Objective Response Rate

A total of 18 studies reported data on ORR, comprising 10 two-arm studies (including 2 RCTs) on probiotics and 8 studies on FMT (comprising 1 RCT and 7 non-randomized single-arm studies). For the comparative analysis (Fig. 2A), which was restricted to two-arm studies, a random-effects model showed that probiotic intervention was associated with a higher ORR (pooled OR = 1.59, 95% CI: 1.12–2.25, *P* = 0.010), with moderate heterogeneity (*I*² = 50%). The FMT subgroup included only one RCT, with OR = 4.00 (95% CI: 0.43–37.11, *P* = 0.22), which was not statistically significant. The overall pooled OR was 1.62 (95% CI: 1.15–2.28, *P* = 0.006), with moderate heterogeneity (*I*² = 46%) and no significant difference between subgroups (*P* = 0.42). Given that the FMT estimate is based on a single RCT, the result for this subgroup should be interpreted with caution and cannot support robust or generalizable conclusions.

Regarding the descriptive analysis of absolute event rates (Fig. 2B), which included data from both single-arm and two-arm studies, the pooled ORR was 50% (95% CI: 0.40–0.60) in the probiotics group and 35% (95% CI: 0.19–0.51) in the FMT group (pooled test *P* = 0.220), indicating no statistically significant difference. Neither subgroup analysis (probiotics: *P* = 0.995; FMT: *P* = 0.078) nor analysis of the total population (*P* = 0.199) reached statistical significance. Although the pooled OR suggests a beneficial effect, the high heterogeneity (*I*² > 80%) across studies and the absence of significant subgroup effects (*P* = 0.42) warrant caution regarding the consistency and generalizability of this estimate. Importantly, the FMT evidence is predominantly derived from single-arm or early-phase studies with limited sample sizes, and should therefore be considered hypothesis-generating rather than confirmatory. Moreover, these pooled proportions provide only absolute event estimates for each group and do not support valid direct cross-group comparisons for evaluating relative treatment efficacy due to the heterogeneous study designs from which they originate. (Fig. 2A-B)

### Disease control rate (DCR)

**Fig. 3:**
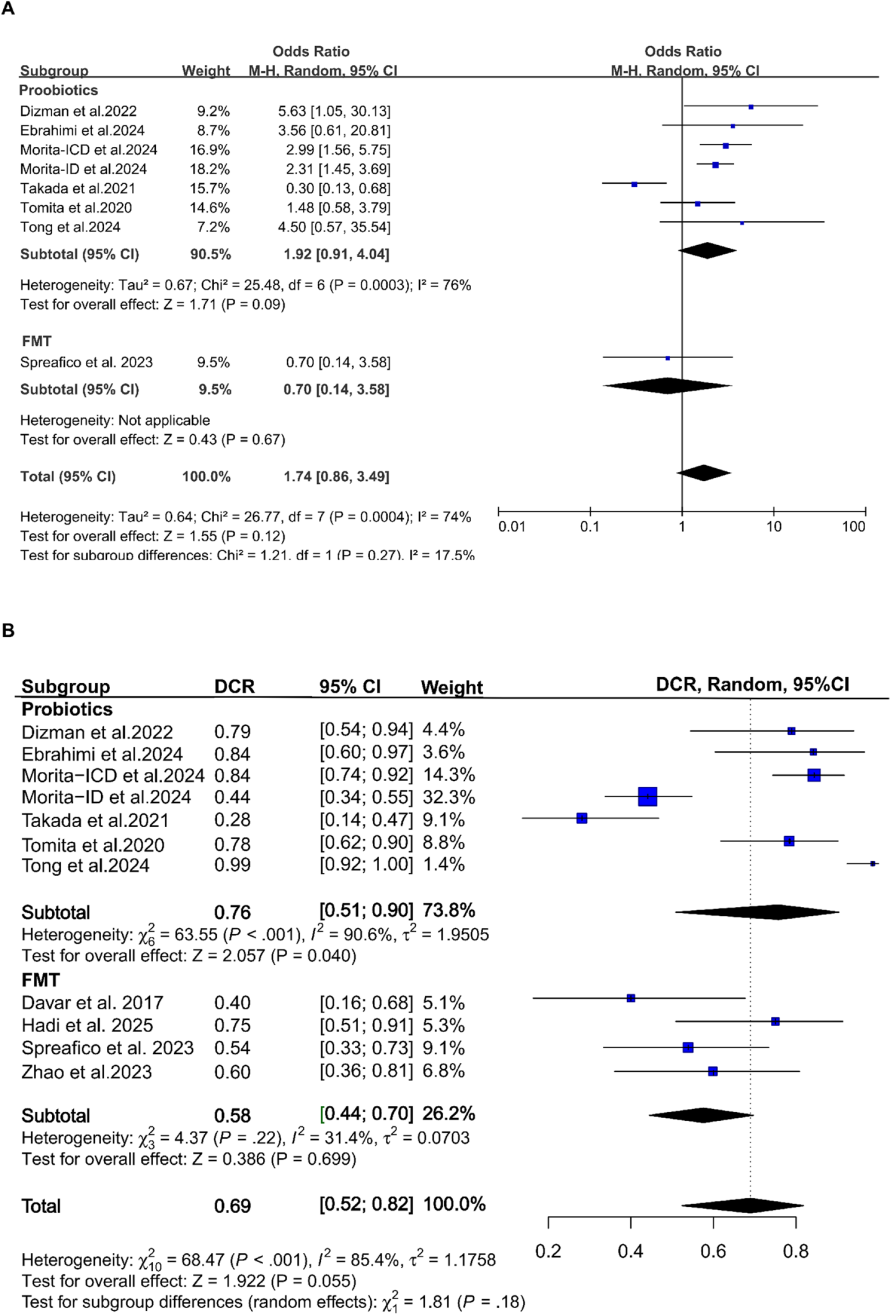
**A**: Forest plot of comparative analysis: Pooled OR for DCR in patients receiving ICIs with probiotics or FMT versus control (based on two-arm studies only). **B**: Forest plot of descriptive analysis: Pooled event rates (proportions) of DCR in patients receiving ICIs with probiotics or FMT (including both single-arm and two-arm studies), Note: These pooled proportions are derived from both single-arm and two-arm studies and should not be interpreted as measures of comparative effectiveness between interventions. Studies are grouped by intervention type and sorted alphabetically by first author’s surname and then by publication year within each group. Abbreviations: FMT, Fecal Microbiota Transplantation; ICIs, Immune Checkpoint Inhibitors; OR, Odds Ratio; DCR, Desease Control Rate

A total of 11 studies reported data on the DCR, including 7 two-arm studies on probiotics (including 2 RCTs) and 4 studies on FMT (comprising 1 RCT and 3 single-arm studies). For the comparative analysis (Fig. 3A), which was restricted to two-arm studies, a random-effects model showed that patients receiving either probiotics or FMT in addition to ICIs exhibited a non-significant trend toward improved DCR compared to control (pooled OR = 1.74, 95% CI: 0.86–3.49, *P* = 0.12), with high heterogeneity across studies (*I*² = 74%). In subgroup analysis based on intervention type, the probiotic subgroup showed a point estimate favoring improved DCR (OR = 1.92, 95% CI: 0.91–4.04, *P* = 0.09), while the FMT subgroup had an OR of 0.70 (95% CI: 0.14–3.58, *P* = 0.67), an estimate derived from limited evidence that warrants cautious interpretation. There was no significant difference between subgroups (*P* = 0.27).

Regarding the descriptive analysis of absolute event rates (Fig. 3B**)**, which included data from both single-arm and two-arm studies, the pooled DCR was 76% (95% CI: 0.51–0.90; *P* = 0.040 for the pooled proportion) in the probiotic subgroup, despite with very high heterogeneity (*I*² = 90.6%, *P* < 0.001). The FMT subgroup had a DCR of 58% (95% CI: 0.44–0.70, *P* = 0.699), with moderate heterogeneity (*I*² = 31.4%, *P* = 0.22). The overall pooled DCR of 69% (95% CI: 0.52–0.82, *P* = 0.055), with very high heterogeneity across studies (*I*² =85.4%, *P* < 0.001). No statistically significant difference was found between subgroups in the random-effects model (*P* = 0.18). Crucially, these pooled proportions provide only absolute event estimates for each group. Due to the heterogeneous study designs from which they originate, direct cross-group comparisons to evaluate relative treatment efficacy are not valid. (Fig. 3A-B)

### Progression-free survival(PFS)

**Fig. 4:**
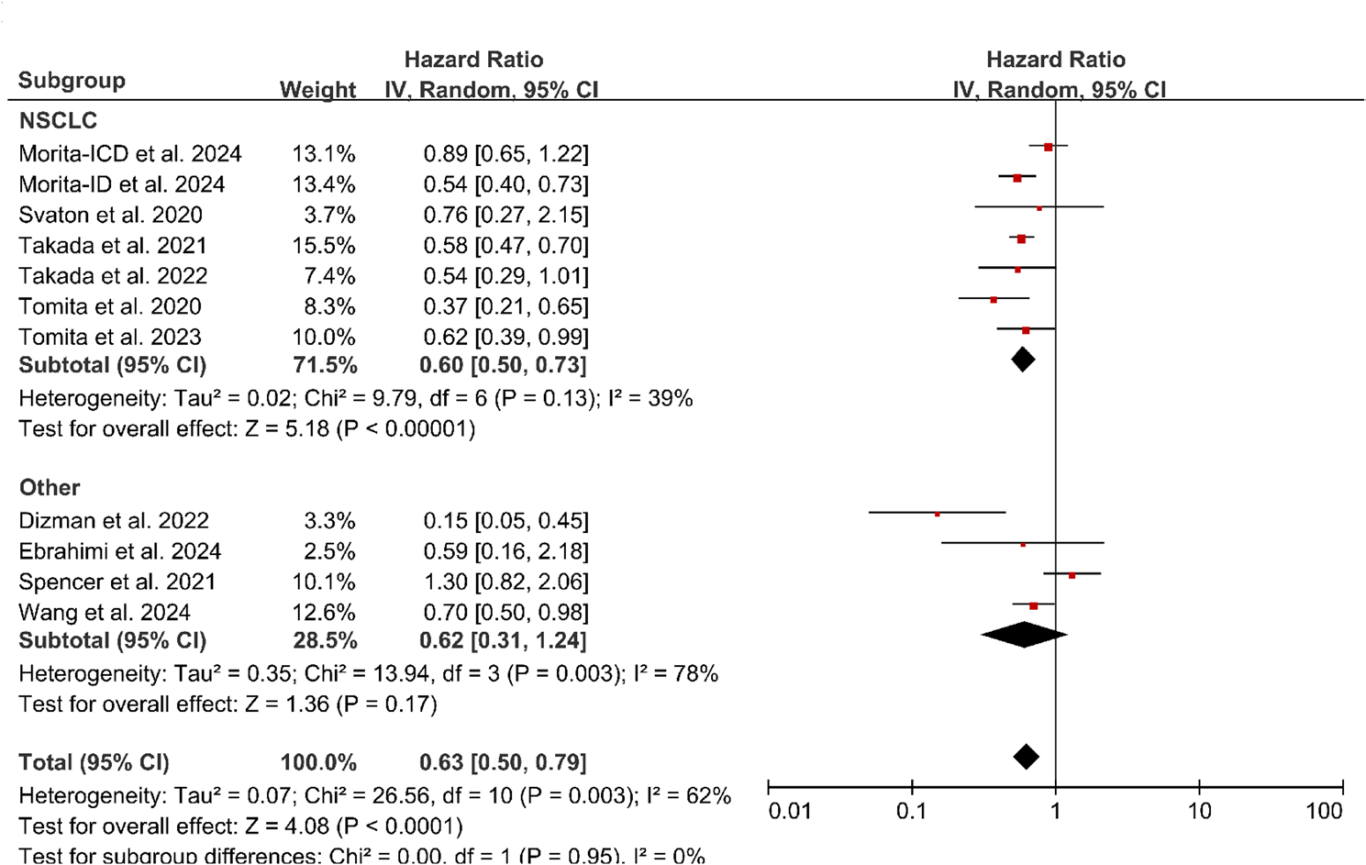
Forest plot of the pooled HR for PFS in patients receiving probiotics during cancer immunotherapy. Note: Subgroups are stratified by tumor type: NSCLC and other cancer types (including melanoma, mRCC, SCLC, HCC, CRC, and GC). Analysis based on two-arm comparative studies only. Abbreviations: CRC, Colorectal Cancer; GC, Gastric Cancer; HR, Hazard Ratio; mRCC, Metastatic Renal Cell Carcinoma; NSCLC, Non-Small Cell Lung Cancer; SCLC, Small Cell Lung Cancer

A total of 13 studies reported data on PFS, all of which focused on probiotic interventions, including11 two-arm comparative studies and 2 RCTs. Under the random-effects model, probiotic intervention was associated with a reduced risk of disease progression or death (pooled HR = 0.63, 95% CI: 0.50–0.79, *P* < 0.0001). High heterogeneity was observed across studies (*I*² = 62%). Subgroup analysis indicated that in patients with NSCLC, probiotic use was associated with improved PFS (HR = 0.60, 95% CI: 0.50–0.73, *P* < 0.00001), with moderate heterogeneity (*I*² = 39%). In contrast, the effect was not statistically significant in other tumor types (HR = 0.62, 95% CI: 0.31–1.24, *P* = 0.17), with very high heterogeneity (*I*² = 78%). Given the substantial heterogeneity within this subgroup, the pooled estimate for non-NSCLC tumors should be interpreted with caution. The “Other” tumor subgroup comprised 4 studies and included the following cancer types: melanoma, metastatic renal cell carcinoma (mRCC), small cell lung cancer (SCLC), hepatocellular carcinoma (HCC), colorectal cancer (CRC), and gastric cancer (GC). Importantly, the difference between the NSCLC and Other subgroups was not statistically significant (*P* = 0.95), with no heterogeneity observed between subgroups (*I*² = 0%). This suggests that the apparent subgroup-specific effects should not be overinterpreted. (Fig. 4A)

### Overall survival (OS)

**Fig. 5:**
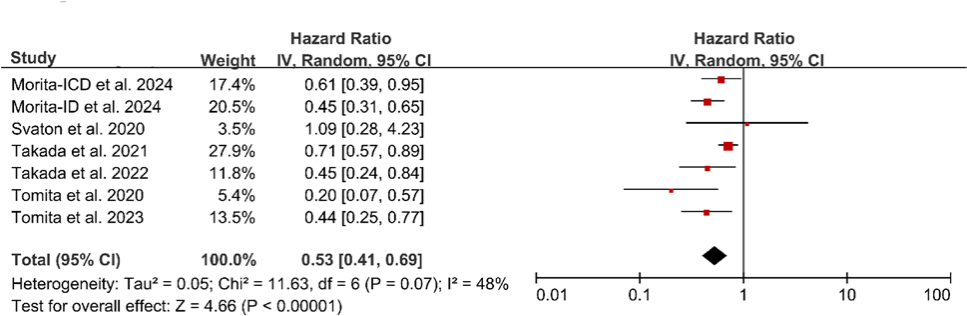
Forest plot of the HR for OS in patients receiving probiotics during cancer immunotherapy. **Note**: Analysis is based on 7 two-arm comparative studies of probiotic interventions. **Abbreviations**: HR, Hazard Ratio

A total of 7 studies, all focusing on probiotic interventions and designed as two-arm comparative studies, reported data on OS. Under the random-effects model, probiotic intervention was associated with a reduced risk of death (pooled HR = 0.53 (95% CI: 0.41–0.69, *P* < 0.00001). Moderate heterogeneity was observed across studies (*I*² = 48%). This pooled estimate corresponds to a 47% reduction in the hazard of death associated with probiotic use during ICI therapy. (Fig. 5)

### Publication bias and sensitive analysis

Regarding the assessment of publication bias, the results of Begg’s and Egger’s tests are summarized in Supplementary Table S1. A notable exception was observed in the PFS analysis, where Egger’s test indicated statistically significant asymmetry (*P* = 0.038), suggesting the possibility of mild publication bias for this outcome. All other Begg’s and Egger’s tests showed no significant publication bias (*P* > 0.05) in the meta-analysis. To evaluate the potential impact of this bias and to assess the robustness of the pooled estimates, sensitivity analyses were conducted. The corresponding funnel plots (Supplementary Figures S2A–F) were inspected; while they primarily serve as visual aids for detecting asymmetry, their consistency across outcomes and the lack of an overtly skewed pattern for PFS provide supportive, albeit not definitive, evidence that no single study exerted a disproportionate influence on the overall results. Considering the significant Egger’s test for PFS, the pooled HR for this outcome should be interpreted with a degree of caution. Nonetheless, the overall body of evidence from the sensitivity analyses suggests that the main findings of this meta-analysis are relatively reliable and stable.

## Discussion

While both probiotics and FMT showed favorable trends, the evidence base for probiotics is notably more robust, comprising multiple cohort studies and RCTs. In contrast, FMT evidence remains largely exploratory, derived from early-phase, single-arm trials with inherent risks of bias. With respect to tumor response, patients receiving microbiota-directed interventions (probiotics and FMT) were associated with an increased ORR (pooled OR = 1.62, 95% CI: 1.15–2.28, *P* = 0.006), while the DCR also demonstrated a trend toward improvement that did not reach statistical significance (pooled OR = 1.74, 95% CI: 0.86–3.49, *P* = 0.12). Considerable heterogeneity was observed for these pooled estimates (*I*² = 46% for ORR and 74% for DCR). In terms of long-term survival outcomes, probiotic supplementation was associated with a reduced risk of disease progression or death, as indicated by a longer PFS (HR = 0.63, 95% CI: 0.50–0.79, *P* < 0.0001) and OS (HR = 0.53, 95% CI: 0.41–0.69, *P* < 0.00001). In subgroup analysis, the point estimate for PFS was more favorable and statistically significant in patients with NSCLC (HR = 0.60, 95% CI: 0.50–0.73, *P* < 0.00001), whereas the point estimate in other tumor types did not reach statistical significance (HR = 0.62, 95% CI: 0.31–1.24, *P* = 0.17). However, the difference between the NSCLC and other tumor subgroups was not statistically significant (*P* = 0.95), and the high heterogeneity observed in the latter subgroup (*I*² = 78%) warrants cautious interpretation.

Moreover, the number of studies and sample size in the FMT group (8 studies, 144 patients) were considerably smaller than those in the probiotic group (13 studies, 3,130 patients), resulting in wider CI. Nevertheless, the point estimates for FMT were consistently favorable and aligned in direction with those of probiotics. This concordance suggests a shared underlying mechanism mediated by the gut-microbiota axis, through which both intervention modalities appear to facilitate a more effective response to ICIs, despite via distinct mechanistic approaches to microbial modulation. However, given the preliminary nature of FMT studies, these findings should be regarded as hypothesis-generating rather than confirmatory.

Though the immunity-microbiota interplay traverses the oral-gut-microbiota axis from its origin in the oral cavity [35], the gut is the dynamic hub of systemic immune adaptation, utilizing its vast interface and complex regulatory network to mobilize immune cells for inter-organ trafficking and to coordinate broad-spectrum immune responses [36, 37]. Such systemic influence is further evidenced by the operations of the microbiota-gut-brain [38, 39] and microbiota-gut-liver axes [40]. Preclinical studies suggest that microbiota-shaped cellular trajectories from the gut can mold peripheral immunity [41], providing a mechanistic rationale for how probiotics and FMT might enhance ICI efficacy.

Mechanistically, distinct pathways are proposed: In immune regulation, specific probiotic strains may promote CD8⁺/CD4⁺ T-cell responses [6, 42], whereas FMT holistically reshapes the beneficial microbial network to systemically modulate the immune microenvironment [30, 31]. Metabolically, probiotics (e.g., CBM588) and FMT-restored bacteria (e.g., *Ruminococcaceae*) produce SCFAs from dietary fiber, which can enhance dendritic cell function and promote T-cell differentiation in models [21]. Regarding barrier and inflammation, probiotics may reinforce intestinal integrity (e.g., VSL#3 proteins in vitro) [43], while FMT in mice restores beneficial taxa like *Akkermansia muciniphila (A. muciniphila)* [44]. As a next-generation probiotic, *A. muciniphila*—particularly via its outer membrane protein Amuc_1100—thickens the mucus layer, further suppressing inflammation and potentially improving response to immunotherapy [45]. Thus, a synergistic model is hypothesized: FMT could broadly reset the microbial ecosystem, from which key beneficial bacteria (e.g., A. muciniphila) could be isolated and and developed into targeted probiotic. However, that these mechanistic insights are derived predominantly from preclinical or indirect clinical evidence, forming plausible hypotheses rather than established patient-level mechanisms.

As research advances, the clinical value of probiotics in immunotherapy is gradually being established, though inconsistencies persist across studies due to limitations in early-phase research [46, 47]. In line with this, the recent meta-analysis by Zhao et al. [48], which pooled data from 13 studies (14 cohorts, 3,142 participants), provides further support for the association of probiotic use with comprehensive clinical benefits in patients receiving ICIs. Specifically, probiotic supplementation was associated with improved OS (HR = 0.58, *P* < 0.001) and PFS (HR = 0.66, *P* < 0.001), as well as enhanced ORR (OR = 1.75, *P* = 0.001) and DCR (OR = 1.93, *P* = 0.002). Benefits were most pronounced in NSCLC patients (OS: HR = 0.51; ORR: OR = 1.83; DCR: OR = 2.21; all *P* < 0.001). Crucially, subgroup analysis confirmed for the first time at the meta-analysis level that probiotics effectively counteracted the negative impact of prior antibiotic exposure in NSCLC patients, yielding significant survival benefits for this specific subgroup (OS: HR = 0.45, *P* < 0.001; PFS: HR = 0.48, *P* < 0.001).

In contrast to the growing body of evidence on probiotics, high-quality evidence for FMT remains limited. A study by Lin et al. [49], which included 10 studies (only 3 of which were two-arm trials), reported a pooled ORR of 43% (95% CI: 0.35–0.51) but didn’t perform pooled analyses for PFS or OS. Their exploratory subgroup analysis suggested higher ORR with combination anti-PD-1 and anti-CTLA-4 therapy (60%) compared to anti-PD-1 monotherapy (37%, *P* = 0.01). It is important to clarify that study by Dizman et al. [15], which utilized the probiotic CBM588 as its intervention, has been included in meta-analyses of both FMT (Lin et al., 2025) and probiotics (Zhao et al., 2025). Given the nature of its intervention, we have assigned it to the probiotic cohort in the present analysis.

This study aims to systematically evaluate the roles of probiotics and FMT in enhancing the efficacy of cancer immunotherapy, with updates and standardization of key data. To ensure accuracy, we re-examined original data sources and excluded the SER-401 study by Glitza et al. [50] due to its small sample size and significant pandemic-related disruptions. Updated survival data from the single-arm FMT trial (Hadi et al.) [34] were incorporated, with median PFS and OS (now at 29.6 months and 52.8 months), respectively. Results suggest a potential association between both probiotic and FMT interventions and improved clinical outcomes of ICIs. Critically, the pooled ORR and DCR are descriptive event rates, not comparative efficacy measures, as they derive from studies of heterogeneous design—a limitation rooted in the evidence base itself, where only 3 of the 22 included studies were RCTs, and high-quality two-arm trials in FMT research were particularly scarce, potentially introducing selection bias.

In the PFS analysis, the non-NSCLC tumor subgroup showed high heterogeneity (*I*^2^ =78%), while the NSCLC subgroup exhibited relatively low heterogeneity (*I*^2^ =39%), suggesting that tumor type may play a differential role in the effect of probiotics on PFS. However, since the between-subgroup difference was not statistically significant, this observation should be interpreted with caution. while unobserved confounders such as variations in antibiotic [10, 14, 16, 22, 23, 29, 31, 32] combination drug (e.g., Fruquintinib) [28, 29] use may further contribute to heterogeneity. Significant variations in study design, patient baseline characteristics, intervention protocols, and outcome assessments also led to moderate-to-high heterogeneity. Although a random-effects model was applied, results should be interpreted with caution, and generalizability remains limited. Notably, this unexplained heterogeneity undermines confidence in the pooled estimates. Furthermore, it suggests that the observed effects may not be uniformly generalizable across clinical settings. Therefore, while the pooled event rates offer a useful summary of absolute responses, future high-quality, head-to-head randomized controlled trials are warranted to directly compare the relative efficacy of these microbial interventions.

## Conclusion

This meta-analysis, which integrated 22 clinical studies involving 3,274 patients, demonstrated that both probiotics and FMT, as adjunctive therapies, are associated with improved outcomes in patients treated with ICIs. These associations were reflected in ORR, DCR, PFS, and OS. However, the evidence base differs markedly between the two interventions. Probiotics are supported by more robust evidence from multiple cohort studies and RCTs, indicating more consistent clinical applicability. In contrast, the evidence for FMT remains preliminary and is derived predominantly from early-phase, single-arm studies; therefore, its comparative efficacy and clinical applicability are less certain. The findings underscore the potential synergistic value of gut microbiota modulation in immunotherapy, while also highlighting the current scarcity of high-quality RCTs (particularly for FMT) and the presence of inter-study heterogeneity. Future efforts should focus on large-scale, rigorously designed clinical studies, including head-to-head comparative trials, to advance personalized and precise gut microbiota-based strategies.

## Supplementary Information

Supplementary Material 1

Supplementary Material 2

## Data Availability

All data generated or analyzed during this systematic review and meta-analysis are included in this published article and its supplementary information files. The original datasets supporting the conclusions of this article were derived from the studies cited in the reference list, all of which are publicly available through their respective publishers.

## Abbreviations

*A. muciniphila*: *Akkermansia muciniphila*
CBM588: *Clostridium butyricum* MIYAIRI 588
CD8⁺ T cells: Cluster of Differentiation 8 Positive T Cells
CR: Complete Response
CRC: Colorectal Cancer
CTLA-4: Cytotoxic T-Lymphocyte-Associated Protein 4
DCR: Disease Control Rate
FMT: Fecal Microbiota Transplantation
GC: Gastric Cancer
HR: Hazard Ratio
ICIs: Immune Checkpoint Inhibitors
IL-12: Interleukin-12
mRCC: Metastatic Renal Cell Carcinoma
NOS: Newcastle-Ottawa Scale
NSCLC: Non-Small Cell Lung Cancer
ORR: Objective Response Rate
OS: Overall Survival
OSCC: Oral Squamous Cell Carcinoma
PD-1: Programmed Cell Death Protein 1
PD-L1: Programmed Death-Ligand 1
PFS: Progression-Free Survival
PR: Partial Response
RCTs: Randomized Controlled Trials
ROB 2.0: Cochrane Risk of Bias 2.0 Tool
ROBINS-I: Risk of Bias in Non-Randomized Studies of Interventions
SCFAs: Short-Chain Fatty Acids
SCLC: Small Cell Lung Cancer
SD: Stable Disease
TH1: T Helper 1
TME: Tumor Microenvironment
