# Supplementary material for: Modulating the gut microbiome to enhance cancer immunotherapy: a systematic review and Meta-Analysis of probiotics and FMT as adjuncts
Source: BMC Cancer. 2026 Jan 28;26:279. doi: 10.1186/s12885-026-15655-6 (PMC12924409; doi:10.1186/s12885-026-15655-6)
Supplement: Supplementary file 1 — Supplementary Material 1 [file 12885_2026_15655_MOESM1_ESM.pdf]

Supplementary materials

Modulating Gut Microbiome to Enhance Cancer Immunotherapy: A Systematic Review and Meta-Analysis of Probiotics and FMT as Adjuncts

Supplemental Table S1. Search strategy.
Supplemental Table S2A. ROB 2.0 Assessment of Risk of Bias in RCTs.
Supplemental Table S2B. NOS Assessment of Risk of Bias in Cohort Studies.
Supplemental Table S2C. ROBINS-I Assessment of Risk of Bias in Single-arm Interventional Studes.
Supplemental Table S3. The Begg's and Egger's tests.
Supplemental Figure S1A-F. Sensitivity analyses.

Supplemental Table S1. Search strategy.

No.	Search Concept	Search Terms/Phrases
#1	Neoplasms/Tumors	("Neoplasms"[MeSH] OR "Neoplasm"[Title/Abstract] OR "Cancer"[Title/Abstract] OR "Tumor"[Title/Abstract] OR "Malignancy"[Title/Abstract] OR "Oncology"[Title/Abstract])
#2	Probiotics	("Probiotics"[MeSH] OR "probiotic"[Title/Abstract] OR "Lactobacillus"[MeSH] OR "Bifidobacterium"[MeSH] OR "Clostridium butyricum"[MeSH] OR "CBM588"[Title/Abstract] OR "MIYAIRI 588"[Title/Abstract])
#3	FMT	("fecal microbiota transplantation"[MeSH] OR "FMT"[Title/Abstract] OR "fecal microbiota transplantation" OR "fecal transplant" OR "stool transplant"[Title/Abstract] OR "intestinal microbiota transplant"[Title/Abstract] OR "Microbiota Transfer Therapy"[Title/Abstract])
#4	Cancer Immunotherapy	("Immunotherapy"[MeSH] OR "immune checkpoint inhibitor"[Title/Abstract] OR "ICI"[Title/Abstract] OR "PD-1 inhibitor"[Title/Abstract] OR "PD-L1 inhibitor"[Title/Abstract] OR "programmed cell death 1 inhibitor"[Title/Abstract] OR "CTLA-4 inhibitor"[Title/Abstract] OR "ipilimumab"[Title/Abstract] OR "nivolumab"[Title/Abstract] OR "pembrolizumab"[Title/Abstract])
#5	Final Combination (Probiotics)	#1 AND #2 AND #4
#6	Final Combination (FMT)	#1 AND #3 AND #4
#7	Overall Union	#5 OR #6

PubMed search as an example.

Supplemental Table S2A. ROB 2.0 Assessment of Risk of Bias in RCTs.

Trial registration number	Author and Year	Bias arising from the randomization process	Bias due to deviations from intended interventions	Bias due to missing outcome data	Bias in measurement of the outcome	Bias in selection of the reported result	Overall
NCT05122546	Ebrahimi et al.2024	Low	Some Concerns	Low	Some Concerns	Low	Some Concerns
NCT03829111	Dizman et.al.2022	Low	Some Concerns	Low	Some Concerns	Low	Some Concerns
NCT03686202	Spreafico et.al. 2023	Low	Some Concerns	Some Concerns	Some Concerns	Some Concerns	Some Concerns

Supplemental Table S2B. NOS Assessment of Risk of Bias in Cohort Studies.

Author and Year	Representativeness of the exposed cohort	Selection of the non exposed cohort	Ascertainment of exposure	Demonstration that outcome of interest was not present at start of study	Comparability of cohorts on the basis of the design or analysis	Assessment of outcome	Was follow-up long enough for outcomes to occur	Adequacy of follow cohort	NOS Score
Svaton et al.2020	★	★	★	★	★	★	★	☆	7/9
Tomita et al.2020	★	★	★	★	★★	★	☆	☆	7/9
Miura et al.2021	★	★	★	☆	★★	★	☆	☆	6/9
Spencer et al.2021	★	★	★	★	★★	★	☆	☆	7/9
Takada et al.2021	★	★	★	★	★★	★	★	☆	8/9
Takada et al.2022	★	★	★	★	★	★	★	☆	7/9
Tomita et al.2023	★	★	★	★	★★	☆	★	☆	7/9
Morita-ID et al.2024	★	★	★	★	★	★	★	☆	7/9
Morita-ICD et al.2024	★	★	★	★	★	★	★	☆	7/9
Wang et al.2024	★	★	★	★	★	★	★	☆	7/9
Luo et al.2024	★	★	★	★	★★	★	★	☆	8/9
Tong et al.2024	★	★	☆	★	★	★	★	★	7/9

Score Standards: A study can be awarded a maximum of one star for each numbered item within the Selection and Outcome categories. A maximum of two stars can be given for Comparability: Yes (★); No (☆).

Supplemental Table S2C. ROBINS-I Assessment of Risk of Bias in Single-arm Interventional Studes.

Trial registration number	Author and Year	Bias due to confounding	Bias in selection of participants	Bias in classification of interventions	Bias due to deviations from intended interventions	Bias due to missing data	Bias in measurement of outcomes	Bias in selection of the reported result	Overall
NCT03341143	Davar et.al. 2017	Moderate	Low	Low	Moderate	Low	Moderate	Moderate	Moderate
NCT03353402	Baruch et.al. 2017	Moderate	Low	Low	Moderate	Low	Moderate	Moderate	Moderate
NCT04163289	Fernandes et.al. 2020	Moderate	Low	Low	Low	Low	Moderate	Moderate	Moderate
ChiCTR2100046768	Zhao et.al. 2021	Moderate	Moderate	Low	Low	Low	Moderate	Moderate	Moderate
NCT04264975	Kim et al. 2024	Moderate	Moderate	Low	Low	Low	Moderate	Moderate	Moderate
NCT04951583	Duttagupta et al. 2024	Moderate	Low	Low	Low	Low	Moderate	Low	Moderate
NCT03772899	Hadi et al. 2025	Moderate	Moderate	Low	Low	Low	Low	Moderate	Moderate

Supplemental Table S3. The Begg's and Egger's tests.

Outcome	Begg's test	Egger's test	Subgroup outcome	Begg's test	Egger's test
ORR	0.600	0.386	ORR in Probiotics	0.677	0.551
ORR (OR)	0.489	0.546	ORR in Probiotics	0.560	0.663
DCR	0.600	0.753	DCR in Probiotics	0.593	0.851
DCR (OR)	0.749	0.667	DCR in Probiotics	0.593	0.710
PFS (HR)	0.064	0.038	PFS in NSCLC	0.293	0.186
OS (HR)	1.000	0.524	-	-	-

Supplemental Figure S1A-F. Sensitivity analyses.

Figure S1A. ORR

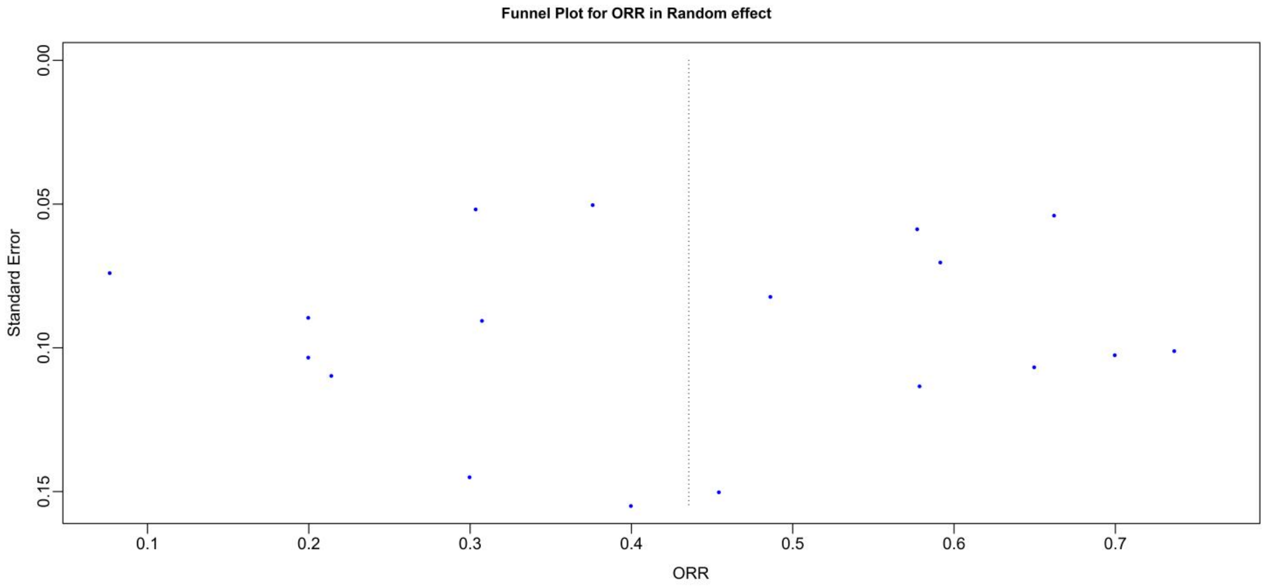

Figure S1B. ORR (OR)

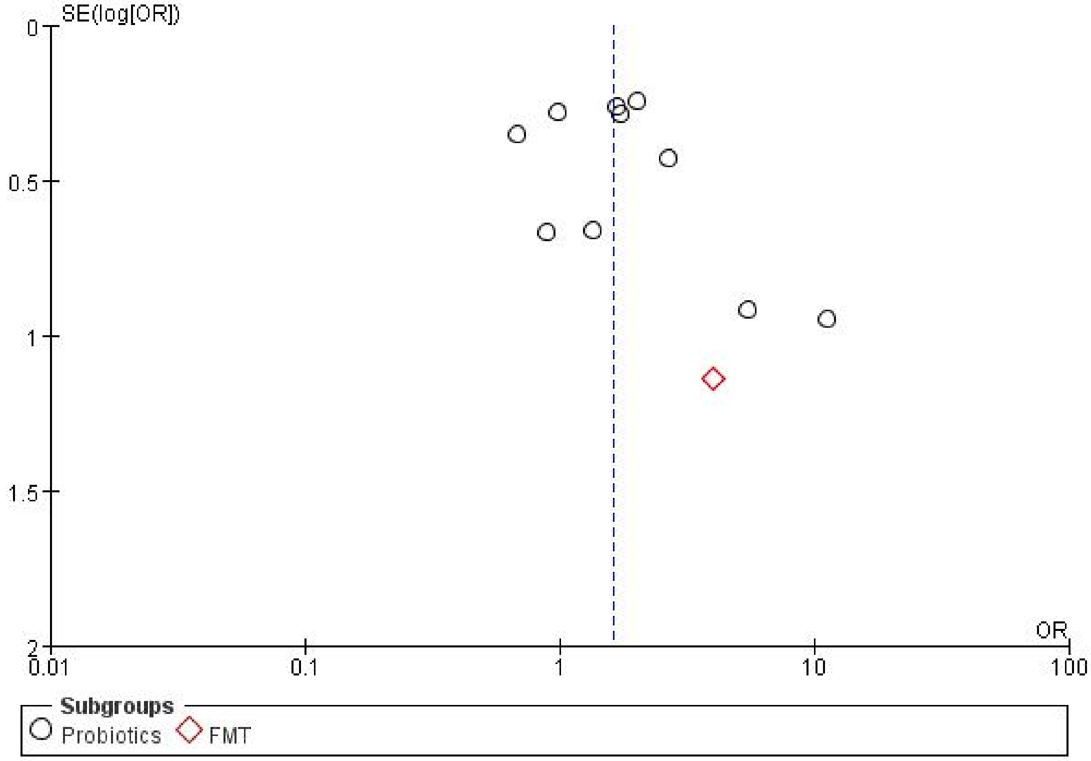

Figure S1C. DCR

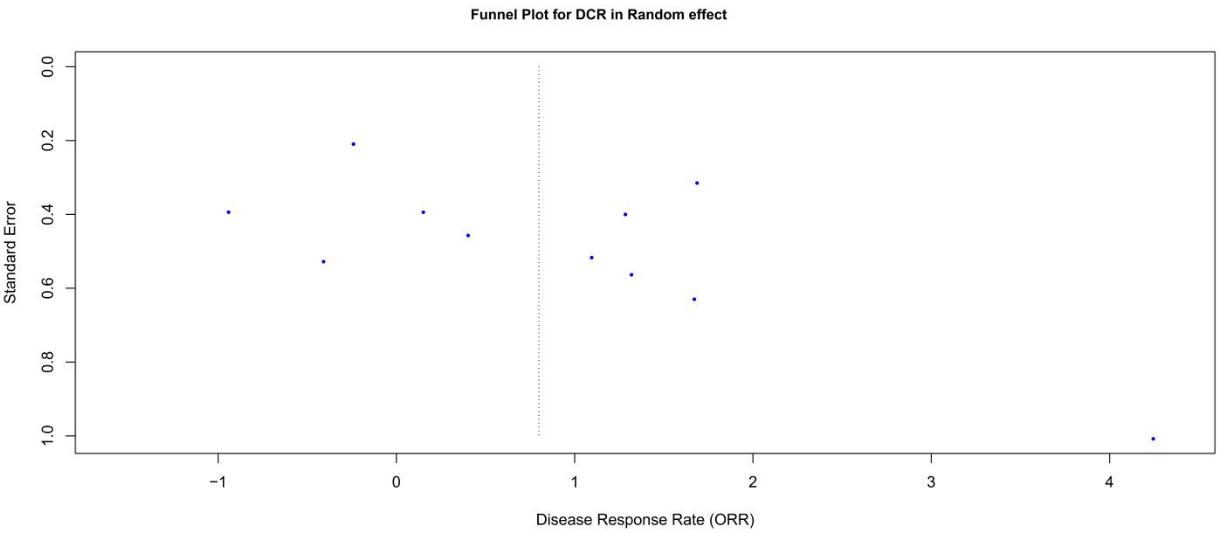

Figure S1D. DCR (OR)

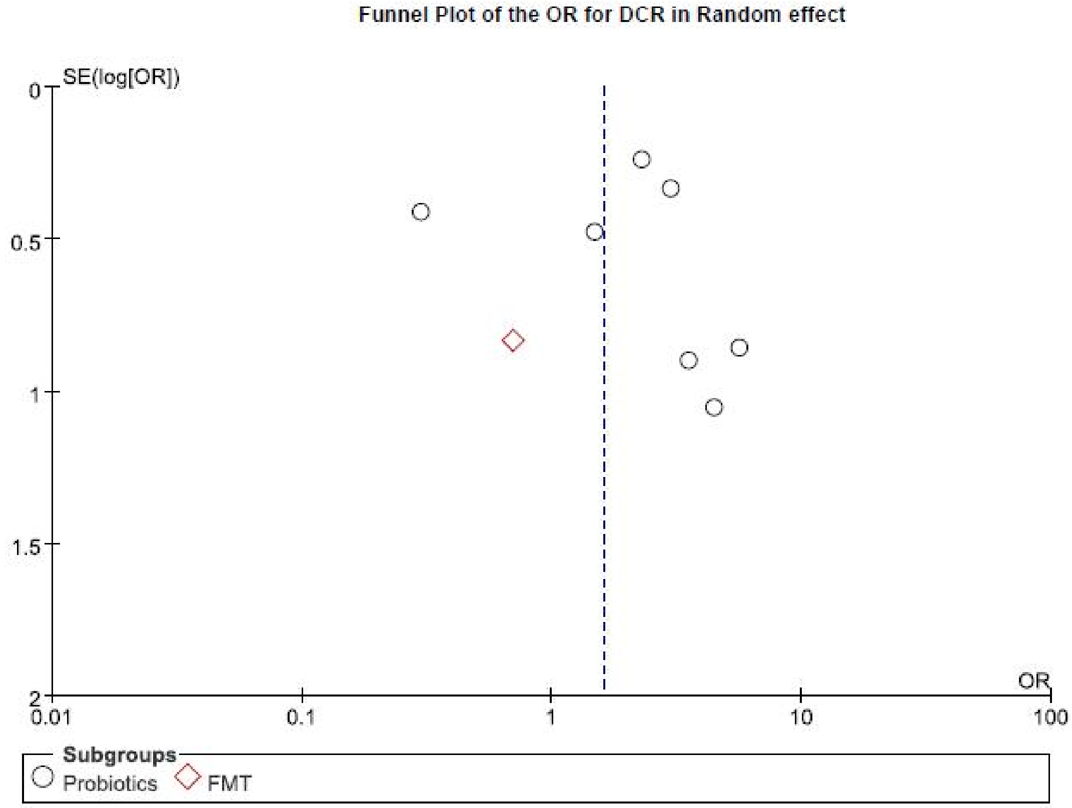

Figure S1E. PFS (HR)

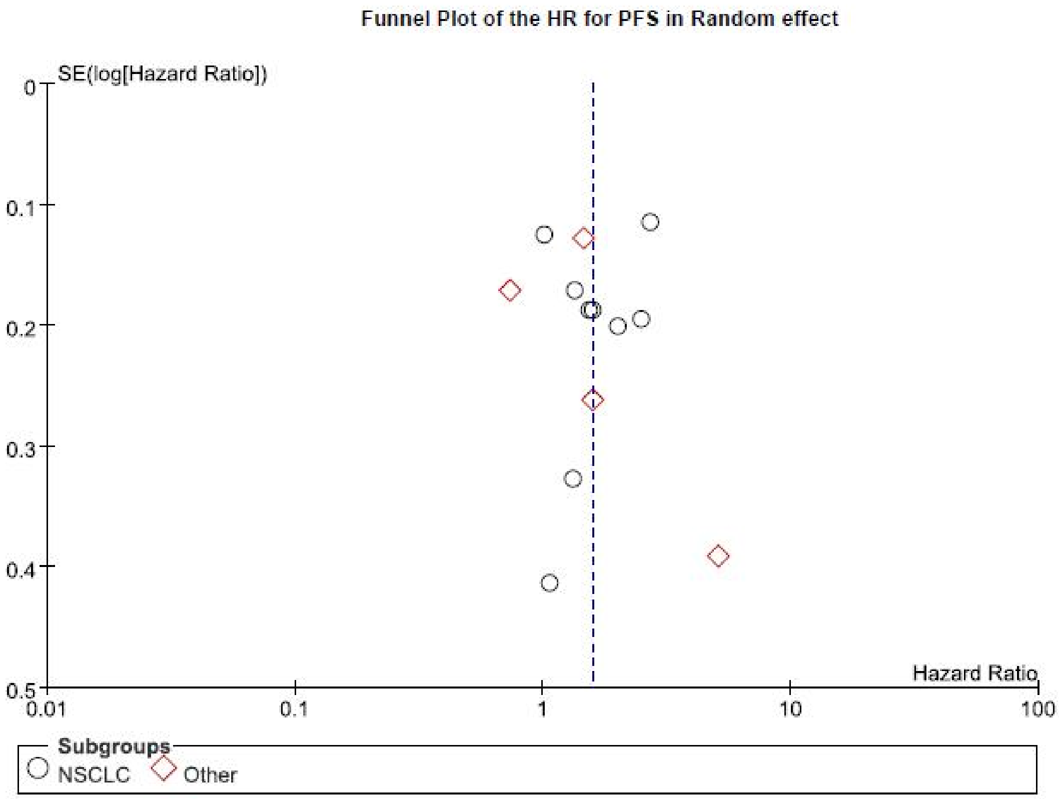

Figure S1F. OS (HR)

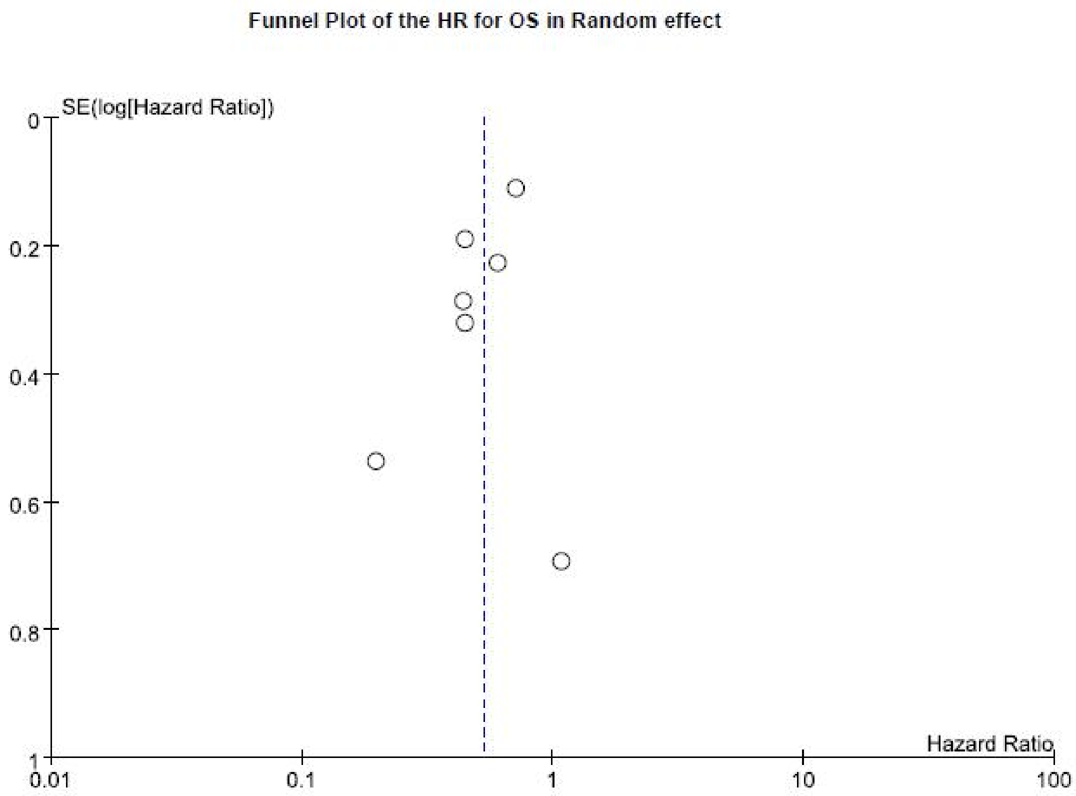

Abbreviations: DCR, Disease Control Rate; HR, Hazard Ratio; NSCLC, Non-Small Cell Lung Cancer; NOS, Newcastle-Ottawa Scale; OR, Odds Ratio; ORR, Objective Response Rate; OS, Overall Survival; PFS, Progression-Free Survival; RCTs, Randomized Controlled Trials; ROB 2.0, Cochrane Risk of Bias 2.0 Tool; ROBINS-I, Risk of Bias in Non-Randomized Studies of Interventions.
