# Supplementary material for: Modulating the gut microbiome to enhance cancer immunotherapy: a systematic review and Meta-Analysis of probiotics and FMT as adjuncts
Source: BMC Cancer. 2026 Jan 28;26:279. doi: 10.1186/s12885-026-15655-6 (PMC12924409; doi:10.1186/s12885-026-15655-6)
Supplement: Supplementary file 2 — Supplementary Material 2 [file 12885_2026_15655_MOESM2_ESM.docx]

**Supplementary materials**

**Modulating Gut Microbiome to Enhance Cancer Immunotherapy: A Systematic Review and Meta-Analysis of Probiotics and FMT as Adjuncts**

**Supplemental Table S1. Search strategy.**

**Supplemental Table S2A. ROB 2.0 Assessment of Risk of Bias in RCTs.**

**Supplemental Table S2B. NOS Assessment of Risk of Bias in Cohort Studies.**

**Supplemental Table S2C. ROBINS-I Assessment of Risk of Bias in Single-arm Interventional Studes.**

**Supplemental Table S3. The Begg’s and Egger’s tests.**

**Supplemental Figure S1A-F. Sensitivity analyses.**

**Supplemental Table S1. Search strategy.**

| No. | Search Concept | Search Terms/Phrases |
| --- | --- | --- |
| #1 | Neoplasms/Tumors | ("Neoplasms"[MeSH] OR "Neoplasm"[Title/Abstract] OR "Cancer"[Title/Abstract] OR "Tumor"[Title/Abstract] OR "Malignancy"[Title/Abstract] OR "Oncology"[Title/Abstract]) |
| #2 | Probiotics | ("Probiotics"[MeSH] OR "probiotic"[Title/Abstract] OR "Lactobacillus"[MeSH] OR "Bifidobacterium"[MeSH] OR "Clostridium butyricum"[MeSH] OR "CBM588"[Title/Abstract] OR "MIYAIRI 588"[Title/Abstract]) |
| #3 | FMT | ("fecal microbiota transplantation"[MeSH] OR "FMT"[Title/Abstract] OR "fecal microbiota transplantation" OR "fecal transplant" OR "stool transplant"[Title/Abstract] OR "intestinal microbiota transplant"[Title/Abstract] OR "Microbiota Transfer Therapy"[Title/Abstract]) |
| #4 | Cancer Immunotherapy | ("Immunotherapy"[MeSH] OR "immune checkpoint inhibitor"[Title/Abstract] OR "ICI"[Title/Abstract] OR "PD-1 inhibitor"[Title/Abstract] OR "PD-L1 inhibitor"[Title/Abstract] OR "programmed cell death 1 inhibitor"[Title/Abstract] OR "CTLA-4 inhibitor"[Title/Abstract] OR "ipilimumab"[Title/Abstract] OR "nivolumab"[Title/Abstract] OR "pembrolizumab"[Title/Abstract]) |
| #5 | Final Combination (Probiotics) | #1 AND #2 AND #4 |
| #6 | Final Combination (FMT) | #1 AND #3 AND #4 |
| #7 | Overall Union | #5 OR #6 |

PubMed search as an example.

| **Supplemental Table S2A. ROB 2.0 Assessment of Risk of Bias in RCTs.** | | | | | | | |
| --- | --- | --- | --- | --- | --- | --- | --- |
| Trial registration number | Author and Year | Bias arising from the randomization process | Bias due to deviations from intended interventions | Bias due to missing outcome data | Bias in measurement of the outcome | Bias in selection of the reported result | Overall |
| NCT05122546 | Ebrahimi et al.2024 | Low | Some Concerns | Low | Some Concerns | Low | Some Concerns |
| NCT03829111 | Dizman et.al.2022 | Low | Some Concerns | Low | Some Concerns | Low | Some Concerns |
| NCT03686202 | Spreafico et.al. 2023 | Low | Some Concerns | Some Concerns | Some Concerns | Some Concerns | Some Concerns |

**Score Standards**: **A study can be awarded a maximum of one star for each numbered item within the Selection and Outcome categories. A maximum of two stars can be given for Comparability: Yes (★); No (☆).**

| **Supplemental Table S2B. NOS Assessment of Risk of Bias in Cohort Studies.** | | | | | | | | | |  |
| --- | --- | --- | --- | --- | --- | --- | --- | --- | --- | --- |
| Author and Year | | Representativeness of the exposed cohort | Selection of the non exposed cohort | Ascertainment of exposure | Demonstration that outcome of interest was not present at start of study | Comparability of cohorts on the basis of the design or analysis | Assessment of outcome | Was follow-up long enough for outcomes to occur | Adequacy of follow cohort | NOS Score |
| Svaton et al.2020 | | ★ | ★ | ★ | ★ | ★ | ★ | ★ | ☆ | 7/9 |
| Tomita et al.2020 | | ★ | ★ | ★ | ★ | ★★ | ★ | ☆ | ☆ | 7/9 |
| Miura et al.2021 | | ★ | ★ | ★ | ☆ | ★★ | ★ | ☆ | ☆ | 6/9 |
| Spencer et al.2021 | | ★ | ★ | ★ | ★ | ★★ | ★ | ☆ | ☆ | 7/9 |
| Takada et al.2021 | | ★ | ★ | ★ | ★ | ★★ | ★ | ★ | ☆ | 8/9 |
| Takada et al.2022 | | ★ | ★ | ★ | ★ | ★ | ★ | ★ | ☆ | 7/9 |
| Tomita et al.2023 | | ★ | ★ | ★ | ★ | ★★ | ☆ | ★ | ☆ | 7/9 |
| Morita-ID et al.2024 | | ★ | ★ | ★ | ★ | ★ | ★ | ★ | ☆ | 7/9 |
| Morita-ICD et al.2024 | | ★ | ★ | ★ | ★ | ★ | ★ | ★ | ☆ | 7/9 |
| Wang et al.2024 | | ★ | ★ | ★ | ★ | ★ | ★ | ★ | ☆ | 7/9 |
| Luo et al.2024 | | ★ | ★ | ★ | ★ | ★★ | ★ | ★ | ☆ | 8/9 |
| Tong et al.2024 | | ★ | ★ | ☆ | ★ | ★ | ★ | ★ | ★ | 7/9 |

| **Supplemental Table S2C. ROBINS-I Assessment of Risk of Bias in Single-arm Interventional Studes.** | | | | | | | | | |
| --- | --- | --- | --- | --- | --- | --- | --- | --- | --- |
| Trial registration number | Author and Year | Bias due to confounding | Bias in selection of participants | Bias in classification of interventions | Bias due to deviations from intended interventions | Bias due to missing data | Bias in measurement of outcomes | Bias in selection of the reported result | Overall |
| NCT03341143 | Davar et.al. 2017 | Moderate | Low | Low | Moderate | Low | Moderate | Moderate | Moderate |
| NCT03353402 | Baruch et.al. 2017 | Moderate | Low | Low | Moderate | Low | Moderate | Moderate | Moderate |
| NCT04163289 | Fernandes et.al. 2020 | Moderate | Low | Low | Low | Low | Moderate | Moderate | Moderate |
| ChiCTR2100046768 | Zhao et.al. 2021 | Moderate | Moderate | Low | Low | Low | Moderate | Moderate | Moderate |
| NCT04264975 | Kim et al. 2024 | Moderate | Moderate | Low | Low | Low | Moderate | Moderate | Moderate |
| NCT04951583 | Duttagupta et al. 2024 | Moderate | Low | Low | Low | Low | Moderate | Low | Moderate |
| NCT03772899 | Hadi et al. 2025 | Moderate | Moderate | Low | Low | Low | Low | Moderate | Moderate |

**Supplemental Table S3. The Begg’s and Egger’s tests.**

| Outcome | Begg’s test | Egger’s test | Subgroup outcome | Begg’s test | Egger’s test |
| --- | --- | --- | --- | --- | --- |
| ORR | 0.600 | 0.386 | ORR in Probiotics | 0.677 | 0.551 |
| ORR (OR) | 0.489 | 0.546 | ORR in Probiotics | 0.560 | 0.663 |
| DCR | 0.600 | 0.753 | DCR in Probiotics | 0.593 | 0.851 |
| DCR (OR) | 0.749 | 0.667 | DCR in Probiotics | 0.593 | 0.710 |
| PFS (HR) | 0.064 | 0.038 | PFS in NSCLC | 0.293 | 0.186 |
| OS (HR) | 1.000 | 0.524 | - | - | - |

**Supplemental Figure S1A-F. Sensitivity analyses.**

**Figure S1A. ORR**


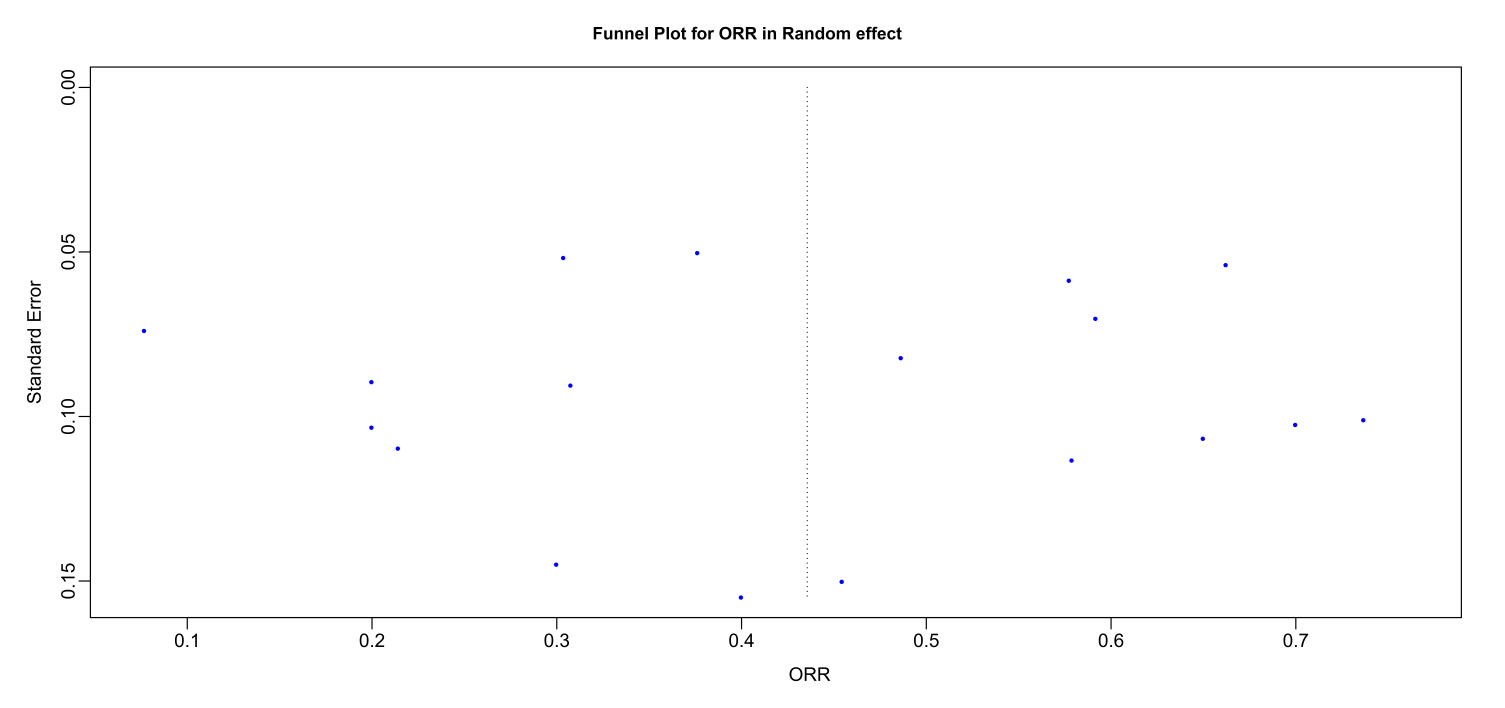


**Figure S1B. ORR (OR)**


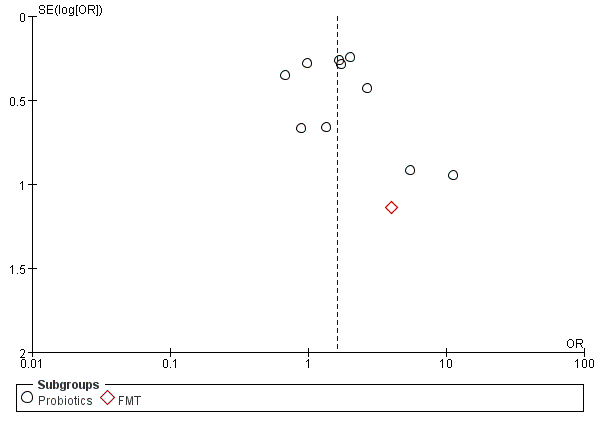


**Figure S1C. DCR**


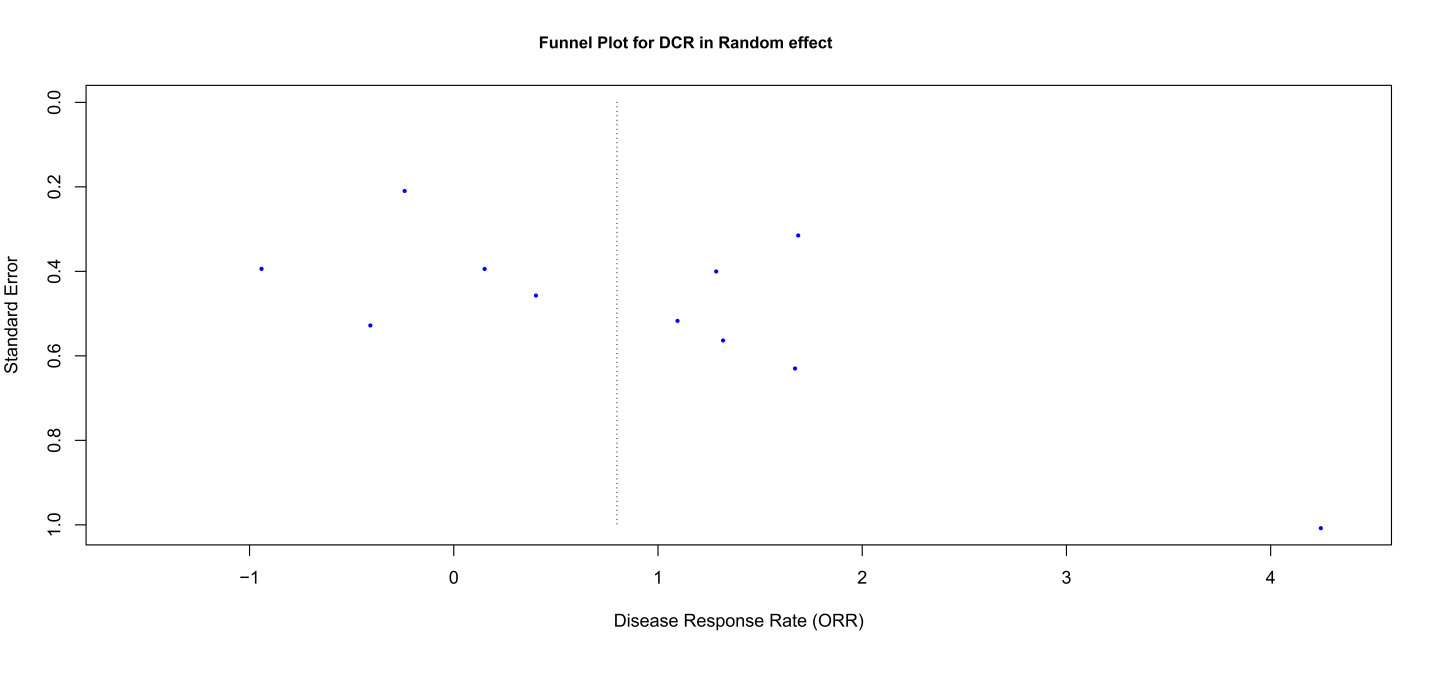


**Figure S1D. DCR (OR)**


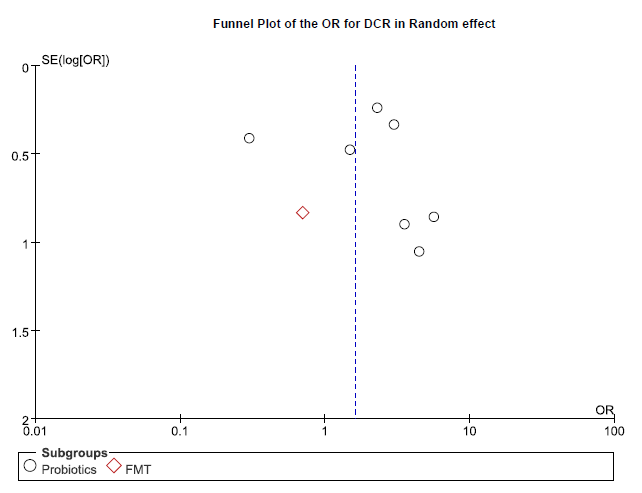


**Figure S1E. PFS (HR)**


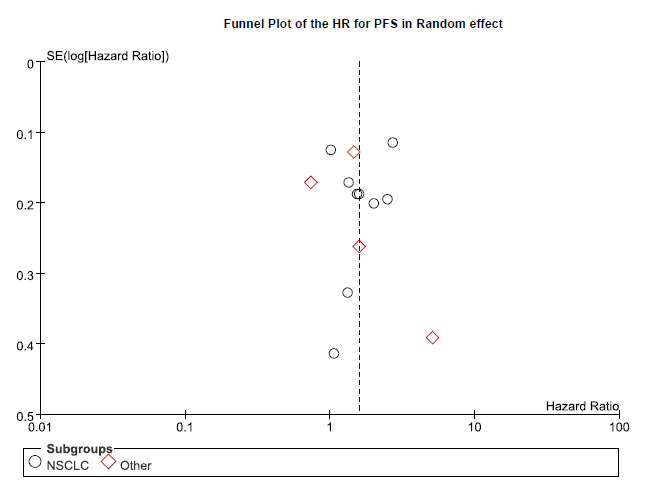


**Figure S1F. OS (HR)**


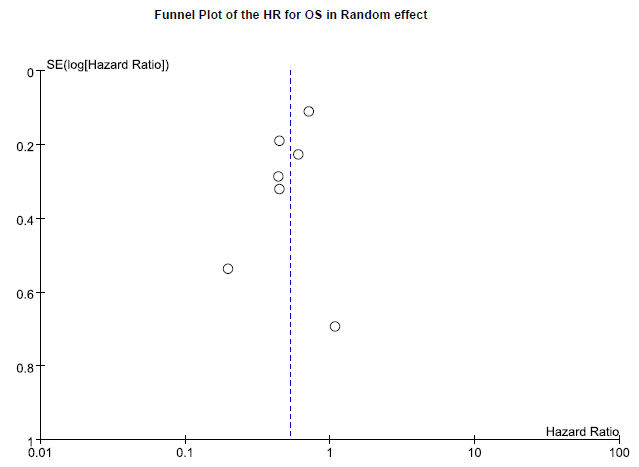


**Abbreviations**: DCR, Disease Control Rate; HR, Hazard Ratio; NSCLC, Non-Small Cell Lung Cancer; NOS, Newcastle-Ottawa Scale; OR, Odds Ratio; ORR, Objective Response Rate; OS, Overall Survival; PFS, Progression-Free Survival; RCTs, Randomized Controlled Trials; ROB 2.0, Cochrane Risk of Bias 2.0 Tool; ROBINS-I, Risk of Bias in Non-Randomized Studies of Interventions.
